# Study on the Mechanism of Enhancing Carbonation Resistance of Wellbore Concrete by Phase-Change in Paraffin Powder

**DOI:** 10.3390/ma19143104

**Published:** 2026-07-20

**Authors:** Zhuang Wang, Tao Han, Xiaopo Wang, Tingting Luo, Qingshan Li, Bing Xue, Yongxiang Lu, Yongsheng Ji

**Affiliations:** 1 Shenhua Group Xinjie Energy Co., Ltd., Ordos 017200, China; 20008279@ceic.com (Z.W.); 20017057@ceic.com (X.W.); greenhill3@163.com (Q.L.); d1141071794@126.com (Y.L.); 2 State Key Laboratory of Intelligent Construction and Healthy Operation and Maintenance of Deep Underground Engineering, China University of Mining and Technology, Xuzhou 221008, China; kdltt@cumt.edu.cn (T.L.); 17712150365@163.com (B.X.); jiyongsheng@cumt.edu.cn (Y.J.)

**Keywords:** paraffin powder, phase transformation film, wellbore concrete, carbonation resistance, enhancement mechanism

## Abstract

The phase transition of paraffin powder mixed in wellbore concrete is generated by baking it, so that the paraffin powder is melted. The influence of baking temperature and paraffin dosage on phase transition degree of paraffin powder and the carbonation resistance of wellbore concrete was studied. Combined with microscopic testing methods such as scanning electron microscopy, the microscopic morphology of phase-transited paraffin films within the cement matrix and the mechanism by which they enhance the carbonation resistance of cement-based materials were analyzed. The results indicate that when baking temperature reaches 80 °C, the paraffin particles in the cement-based material can melt into viscous droplets. Partially melted paraffin particles condense within the internal pores of the cement matrix upon cooling, sealing the transmission pathways of CO_2_ gas into the cement-based material. Fully melted paraffin droplets flow within the internal pores of the cement matrix and, upon condensation, firmly adhere to the pore walls, forming a relatively dense and smooth film. This film isolates CO_2_ gas contact with the cement matrix, significantly improving carbonation resistance of the cement-based material. When the paraffin dosage exceeds 3% and the baking temperature surpasses 100 °C, the phase transition of paraffin powder achieves a fully carbonation-resistant effect.

## 1. Introduction

The mine shaft serves as a critical conduit in the realm of energy mining; thus, the safety and reliability of the shaft are paramount to ensuring its secure operation throughout the entire mining period. Consequently, the design service life of mine shafts is significantly extended. Specifically, the designed service life of shafts at the Xinjie mine in China is projected to exceed one century, approximately 130 years. However, mining operations generate substantial quantities of harmful gases, including carbon monoxide, carbon dioxide, and methane. These gases present significant health hazards to miners, potentially resulting in poisoning or explosive incidents if not discharged promptly. Mine ventilation systems are capable of effectively eliminating these harmful gases, thereby ensuring the safety and well-being of miners [1,2]. The harmful gases circulating through the mine shaft induce significant carbonation of wellbore concrete [3,4]. Concrete carbonation results in the corrosion of the embedded steel reinforcement, thereby directly impacting the durability and overall service life of mine shafts [5].

The hydration of cement within concrete results in the generation of substantial quantities of alkaline substances, including calcium hydroxide and calcium silicate hydrate (C-S-H gel) [6]. Carbon dioxide present in the atmosphere diffuses through the pores of concrete, subsequently reacting chemically with the hydration products of cement, including calcium hydroxide and calcium silicate hydrate. This process is commonly referred to as carbonation [7,8]. The carbonation process substantially decreases the pH value of the solution within the concrete pores [9]. When the pH reaches a critical threshold, the passivation layer on the surface of the embedded steel within the concrete can be compromised [10]. Concrete carbonation results in the corrosion of the reinforcement embedded within the concrete, directly influencing the durability and service life of reinforced concrete structures [11].

Concrete is classified as a porous material [12]. Enhancing the density and reducing the porosity of concrete are critical strategies for preventing the diffusion of CO_2_ gas into the material. Presently, the most prevalent approach involves incorporating mineral admixtures, such as fly ash, or nanomaterials like nano-silica into the concrete matrix [13]. These measures can notably enhance the carbonation resistance of concrete to a certain degree. However, the improvement in carbonation resistance remains marginally significant [14,15]. This level of improvement is insufficient for ensuring the durability of coal mine shafts.

The application of anti-corrosion coatings on concrete surfaces serves to isolate and prevent external atmospheric CO_2_ from penetrating the interior of the concrete, representing a cost-effective and practical durability protection method [16,17,18]. Currently, the conventional protective coatings utilized for concrete surfaces primarily encompass organic coatings [19,20] and inorganic coatings [21,22]. On one hand, organic coatings demonstrate commendable film-forming properties coupled with high adhesion, which collectively yield an effective barrier effect. However, when subjected to aggressive environmental conditions over extended periods, organic coatings are susceptible to degradation and aging, ultimately culminating in a reduced service life and their inability to provide long-term protection against concrete carbonation. Conversely, inorganic coatings are characterized by inferior film-forming and bonding properties, rendering them particularly vulnerable to cracking and peeling from the concrete surface, thereby compromising their ability to effectively isolate CO_2_ from infiltrating the concrete.

Paraffin wax is predominantly composed of alkane compounds, exhibiting a relatively stable chemical structure that renders it less susceptible to reactions with other substances. The anti-aging properties and inherent stability of paraffin wax fulfill the criteria for ensuring long-term durability and protection under ambient conditions [23,24]. Furthermore, paraffin wax possesses a low melting point and remains stable under thermal conditions; it can undergo a phase transition into a fluid characterized by exceptional flowability [25,26,27]. Upon heating and melting, paraffin wax demonstrates a high affinity, strong adhesion, and effective sealing properties in relation to cement-based materials. Liquid paraffin has the capacity to infiltrate and permeate the fine capillaries and cracks within the concrete structure, thereby facilitating the complete sealing of the concrete surface and establishing an effective barrier effect [28,29,30]. When employed as a protective coating for concrete surfaces, it can effectively mitigate the challenges of rapid degradation and the limited service life typically associated with organic coatings. Presently, research on the application of phase-change paraffin wax for carbonation protection in cement-based materials remains limited.

The solid–liquid phase transition temperature range of paraffin wax is relatively narrow. If liquid paraffin is directly sprayed onto the concrete surface, it rapidly condenses upon contact with the cooler concrete. Although this can form a dense wax seal layer on the concrete surface, the adhesion between this condensed layer and the concrete is poor. Additionally, the wax seal layer has low strength and poor toughness, leading to cracking and peeling shortly after application. Thus, paraffin wax fails to achieve the intended durability protection effect. This paper proposes a research approach involving the baking of cement-based materials with internally incorporated paraffin powder to induce phase transition of the paraffin powder into a film, thereby enhancing the concrete’s carbonation resistance. Under external heat source baking, the paraffin powder in the cement-based material melts and liquefies. The liquid paraffin penetrates through the concrete pores and condenses to form a wax seal on the pore walls, creating an integrated wax-sealed protective layer within the concrete. This can effectively avoid the issue of easy peeling of paraffin coatings on the concrete surface. Even under external loads, the paraffin layer cannot be easily peeled off, achieving long-term and effective protection of the concrete’s carbonation resistance.

This study investigates the effects of baking temperature and paraffin dosage on the carbonation resistance and phase transition degree of cement-based materials with paraffin phase transition films by observing the phase transition and melting state of externally incorporated paraffin powder in cement-based materials under baking conditions and measuring their carbonation resistance. Simultaneously, through water contact angle tests and scanning electron microscopy (SEM) analysis, the hydrophobic characteristics of the cement matrix and the microscopic morphology of the internal phase-change paraffin film are examined to explore the protective mechanism by which paraffin phase transition into a film enhances the carbonation resistance of cement-based materials.

## 2. Materials and Tests

### 2.1. Raw Materials

The cementitious material used in the tests was ordinary Portland cement (P·O 42.5) produced by Xuzhou Chengyi Cement Company (Xuzhou, Jiangsu, China). The cement has a density of 3.14 g/cm^3^, a standard consistency water demand of 28.1%, a residue of 1.02% on the 0.08 mm square-hole sieve and a specific surface area of 330 m^2^/kg. The mineral contents of C_3_S, C_2_S, C_3_A, and C_4_AF in the cement are 56.32%, 20.29%, 6.98%, and 10.37%, respectively. Its chemical composition is shown in Table 1. The paraffin used in the tests was fully refined paraffin powder produced by Shijiazhuang Yima Chemical Co., Ltd. (Shijiazhuang, China), with a melting point of 58 °C, its particle size no greater than 37.4 μm, and an oil content of 0.5%. Fine aggregate and water used for molding specimens were standard sand and deionized water, respectively. The CO_2_ gas for carbonation tests was produced by Xuzhou Special Gas Factory (Xuzhou, Jiangsu, China), with a purity higher than 99.5%.

### 2.2. Preparation of Phase-Change Paraffin-Coated Cement-Based Mortar Specimens

With reference to the Method of Testing Cements—Determination of Strength (ISO Method) (GB/T 17671-2021) [31], cement mortar specimens incorporating paraffin powder were prepared by replacing fine aggregate with paraffin powder in equal amounts. The paraffin dosages were 0%, 1%, 3%, 5%, 7% and 10% of the cement mass, corresponding to specimen designations L0, L1, L3, L5, and L7, respectively. The specimens measured 40 mm × 40 mm × 160 mm, with a water–cement ratio of 0.5 and a cement–sand ratio of 1:3. After demolding at 24 h under standard curing conditions (temperature 20 ± 2 °C, relative humidity ≥ 95%), the specimens continued standard curing until reaching 28 days of age.

**Figure 1:**
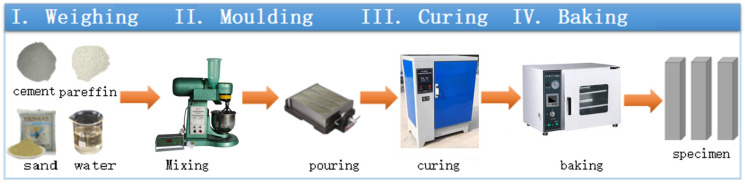
Pretreatment process of phase-change to film cement mortar specimens.

The specimens were placed in an electric thermostatic drying oven and baked at a constant temperature for 3 h to induce phase-change melting of the paraffin particles within the specimens. Each group of specimens was subjected to one of three baking temperatures: 60 °C, 80 °C, and 100 °C, with corresponding designations AX-60 °C, AX-80 °C, and AX-100 °C (where X ∈ {0, 1, 2, 3, 4}). Additionally, a portion of the specimens from group A0 was reserved without baking to serve as the standard-cured reference group for pure cement specimens, designated as L0′. Pretreatment process of phase-change to film cement mortar specimens shows Figure 1.

### 2.3. Test Research Content

#### 2.3.1. Effect of Baking Temperature and Paraffin Dosage on Cement-Based Materials

After the baked mortar specimens of each group had naturally cooled to room temperature, an electronic universal testing machine (Instron, Norwood, MA, USA) was used to measure their compressive strength. This was conducted to investigate the influence of baking temperature and paraffin dosage on the mechanical properties of the phase-change paraffin-coated cement mortar. The compressive strength for each group of specimens was determined by taking the arithmetic mean of three measured values.

#### 2.3.2. Effect of Baking Temperature on Carbonation Resistance of Cement-Based Materials

After the pretreatment of specimens was completed, they were further dried at 65 °C for 48 h. Following the Standard for Test Methods of Long-term Performance and Durability of Ordinary Concrete (GB/T 50082-2009) [32], the treated specimens were placed into a carbonation chamber to initiate carbonation testing. The carbonation ages were set at 3 days, 7 days, and 28 days, respectively, to investigate the carbonation resistance of the phase-change coated cement-based materials.

The specimens were cured in a standard curing room (20 ± 1 °C, relative humidity > 95%) until they reached an age of 28 days, then they were placed in a drying oven at 60 °C for continuous drying for 48 h. The two opposite sides of the specimens served as carbonation surfaces. A straight line was drawn every 10 mm along the length as carbonation depth measurement points, then the carbonation surfaces were sealed with paraffin. The sealed specimens were placed in a carbonation chamber for carbonation, ensuring the spacing between specimens was greater than 50 mm. The parameters of the carbon chamber were set with the CO_2_ concentration at (20 ± 3)%, the temperature at (20 ± 2) °C, and the relative humidity at (70 ± 5)%.

After the carbonation test, the fractured specimens were exposed to air for 90 days. A 1% phenolphthalein solution was then sprayed onto the cross-sections, and the color development on the specimen cross-sections after spraying was recorded by photography. This was conducted to study the carbonation characteristics of the accelerated carbonated phase-change of coated cement-based materials under natural environmental conditions.

#### 2.3.3. Effect of Baking Temperature and Paraffin Dosage on the Phase Transition Degree

After the baked mortar specimens from each group were slowly cooled to room temperature, video microscopy was employed to observe the phase transition and melting state of paraffin on the specimen surfaces and cross-sections at magnification. This was conducted to investigate the influence of baking temperature and paraffin dosage on the phase transition degree of paraffin within the cement-based materials.

#### 2.3.4. SEM Analysis of Paraffin Phase-Change of Coated Cement-Based Materials

(1) Preparation of test specimens

After measuring the water contact angle of each group of cement matrices as described in Section 2.3.4, samples were taken from the interior of the matrix and broken into cubic blocks with side lengths ≤ 5 mm. These blocks were immersed in absolute ethanol for 48 h to terminate hydration, followed by drying in a 60 °C constant-temperature forced-air drying oven for 3–6 h. The surfaces of the blocks were sequentially polished using a Buehler VECTOR automatic variable-speed polishing machine (Buehler, Lake Bluff, IL, USA) and coated with gold via an ion sputter coater (Quorum Technologies, Lewes, UK), thereby preparing the specimens for SEM testing.

(2) SEM observation

A Quanta 250 environmental scanning electron microscope (Thermo Fisher Scientific (FEI), Hillsboro, OR, USA) was used to analyze the microstructure of the test specimens. The samples were secured on the observation stage of the SEM, and observations were conducted within an acceleration voltage range of 5 kV to 10 kV and a probe current range of 1 pA to 2 mA. This study aimed to investigate the microstructural characteristics of the phase-change paraffin within the cement matrix.

## 3. Test Results and Analysis

### 3.1. Effect of Baking Temperature and Paraffin Dosage on Compressive Strength of PPFCM

**Figure 2:**
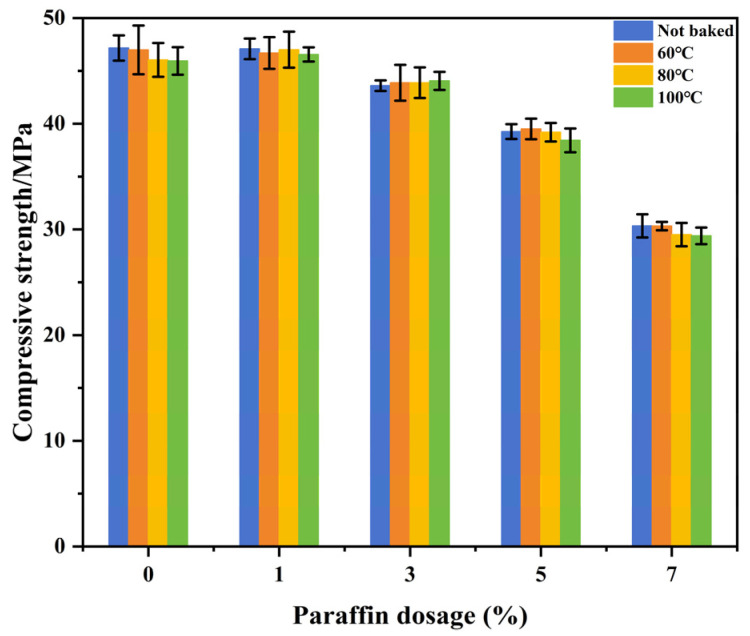
Effect of baking temperature and paraffin dosage on mechanical properties of PPFCM.

The influence of baking temperature and paraffin dosage on the compressive strength of PPFCM is illustrated in Figure 2. The 28-day compressive strength of the standard-cured pure cement mortar specimen (L0′) was 47.31 MPa. The compressive strengths of the baked pure cement mortar specimens, L0-60 °C, L0-80 °C, and L0-100 °C, were 47.55 MPa, 46.63 MPa, and 46.49 MPa, respectively. These results indicate that the compressive strength of the pure cement mortar specimens decreased slightly with increasing baking temperature.

At a paraffin dosage of 1%, the compressive strengths of the PPF mortar specimens (L1-60 °C, L1-80 °C, and L1-100 °C) were 46.98 MPa, 47.12 MPa, and 46.77 MPa, respectively. These results demonstrate that the compressive strength of the mortar containing 1% paraffin exhibited a slight downward trend with increasing baking temperature. Furthermore, compared to the pure cement mortar specimen (L0), the compressive strength at each baking temperature showed a minor reduction (within 2 MPa).

When the paraffin dosage was increased to 3%, the compressive strengths of the PPF mortar specimens (L3-60 °C, L3-80 °C, and L3-100 °C) were 43.88 MPa, 43.75 MPa, and 43.95 MPa, respectively. These values represent a further slight decrease compared to the specimens with 1% paraffin (L1) at each corresponding baking temperature.

As the paraffin dosage further increased to ≥5%, the phase-change film-forming cement mortar specimens L5 (5%) and L7 (7%) exhibited a substantial decline in compressive strength across all baking temperatures, with values falling below 40 MPa. Notably, specimen L7-100 °C (7% paraffin) recorded the lowest compressive strength of 27.53 MPa, representing only 55% of the strength of specimen L0′.

Consequently, the compressive strength of the phase-change film-forming cementitious material demonstrated an initial slight decrease followed by a significant reduction as the paraffin dosage increased, with the critical threshold for substantial strength loss occurring at 5% paraffin dosage. Furthermore, at equivalent paraffin dosage levels, the compressive strength exhibited a mild decreasing trend with rising baking temperature.

### 3.2. Effect of Baking Temperature and Paraffin Dosage on Carbonation Resistance of PPFCM

**Figure 3:**
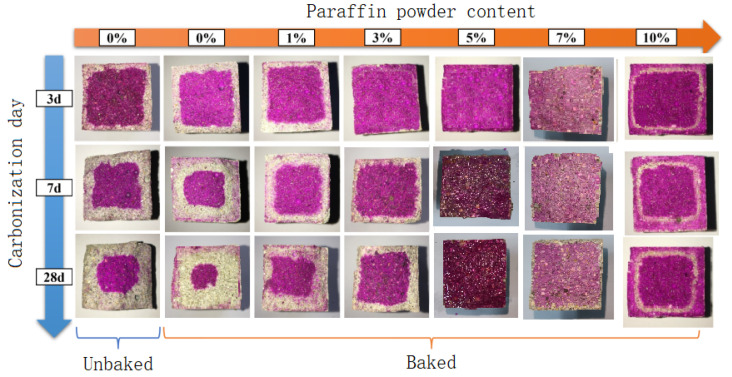
Carbonization depth of cement mortar specimens.

**Figure 4:**
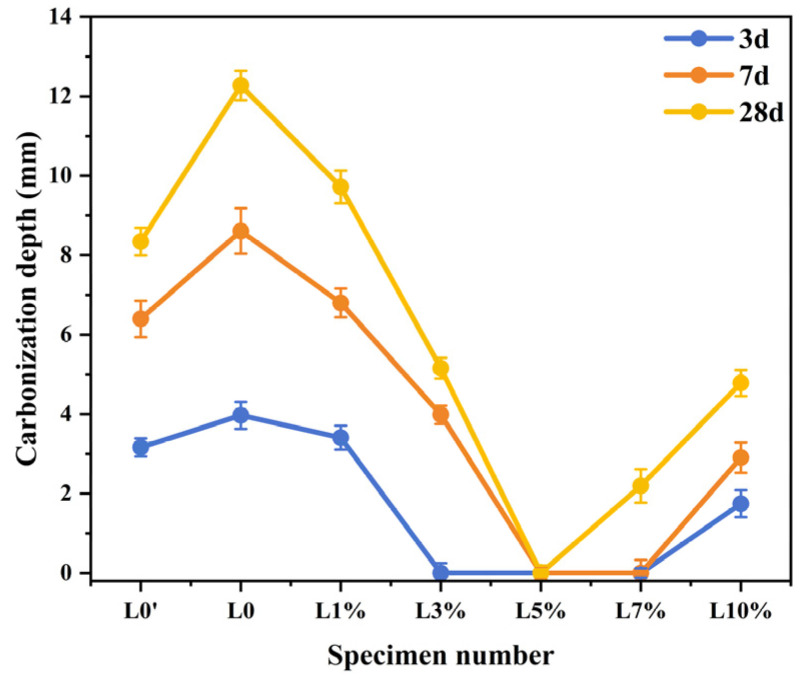
Change of carbonization depth of heat-treated cement mortar specimens.

The change of carbonation depth of specimens at different ages is shown in Table 2 and Figure 3. It can be seen from the figure that the carbonation of specimens L0′ without paraffin powder is more obvious, the carbonation depth gradually increases with the extension of age, and the carbonation depth at 3 d, 7 d and 28 d is 3.23 mm, 6.40 mm and 8.32 mm, respectively. The baking treatment has a more significant effect on the anti-carbonation performance of cement-based materials, and the 3 d, 7 d and 28 d carbonation depth of specimens L0 without paraffin is 3.99 mm, 8.59 mm and 12.25 mm, respectively. When the content of paraffin powder is low, the effect of baking treatment on the anti-carbonation performance of the cement-based material is not obvious; the 3 d, 7 d and 28 d carbonation depths of the specimens L1 with 1% paraffin powder are 3.43 mm, 6.82 mm and 9.70 mm, respectively. With the increase in paraffin powder dosage, the anti-carbonation performance of cement-based materials becomes stronger and stronger, and the carbonation depth of the specimens with paraffin powder is obviously smaller than that of the pure cement specimens (as shown in Figure 4). The greater the addition amount of paraffin powder, the smaller the carbonization depth of cement-based materials. When the addition amount of paraffin reaches 5%, the 28-day carbonization depth of L5 specimens is close to 0. The improvement of the anti-carbonation performance of cement-materials by the paraffin protective layer is very significant.

With a further increase in the paraffin powder content, the carbonation resistance of the cementitious materials slightly decreased. Specimen L7, with 7% paraffin powder content, exhibited slight carbonation to a depth of 2.23 mm at a carbonation age of 28 days. In contrast, specimen L10 with a 10% paraffin powder content, showed a ring-shaped uncolored area on its surface layer at a carbonation age of 3 days. This uncolored closed ring moved inward as the carbonation age extended, and the area it passed through turned pink again under the action of the phenolphthalein indicator. This may be caused by the excessive powder content affecting the hydration of the cement. Therefore, to improve the carbonation resistance of cementitious materials, the paraffin powder content should ideally range from 5% to 7.

### 3.3. Effect of Baking Temperature and Paraffin Dosage on Phase-Change Degree of Paraffin

(1) Specimens surface

**Figure 5:**
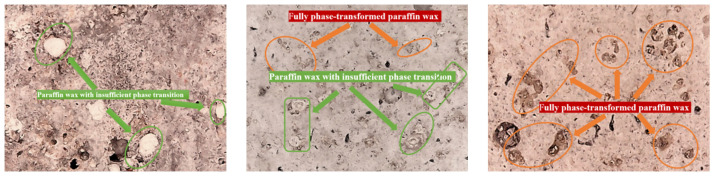
Phase-change melting state of paraffin on the surface of cement mortar.

Figure 5 illustrates the phase-change molten state of paraffin on cementitious composite surfaces. Specimens L5-60 °C, L5-80 °C, and L5-100 °C all exhibit surface-adhered paraffin, indicating phase-change aggregation and exudation to the specimen surfaces that directly provides sealing effects.

These paraffins were white (L5-60 °C), white and dark mixed oil-stained (L5-80 °C) and dark oil-stained (L5-100 °C), respectively. This demonstrates progressive chromatic darkening of PPFCM surface paraffin with elevated curing temperatures.

This shows that the white shape means that the paraffin is not fully melted, and its bonding effect with the cement matrix is poor. The dark oil stains represent that the paraffin wax has been fully phase-transformed and melted. The paraffin wax in this state is difficult to be peeled off by external force and forms a whole with the cement matrix. Therefore, the phase transition degree of paraffin wax on the surface of PPFCM specimen increases with the increase in baking temperature.

(2) Inside specimens

① Baking temperature: 60 °C

**Figure 6:**
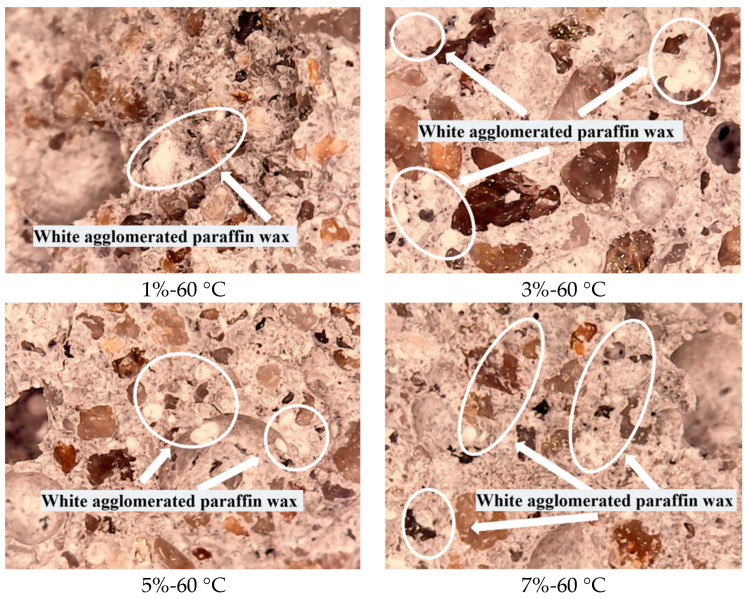
Phase-change melting state of paraffin on the surface of cement mortar under 60 °C.

Figure 6 presents the phase-change molten state (50× magnification) of paraffin within specimens cured at 60 °C. The micrograph reveals whitish paraffin agglomerates embedded in the matrix, with their prevalence increasing proportionally to paraffin dosage. This indicates persistent incomplete phase transition in the bulk material at 60 °C curing temperature, where impaired flowability prevents adequate exudation and diffusion. At higher paraffin dosages, adjacent under-molten domains coalesce into macroscopic whitish agglomerates, resulting in localized protective zones with ineffective film-forming diffusion throughout the matrix.

② Baking temperature 80 °C

**Figure 7:**
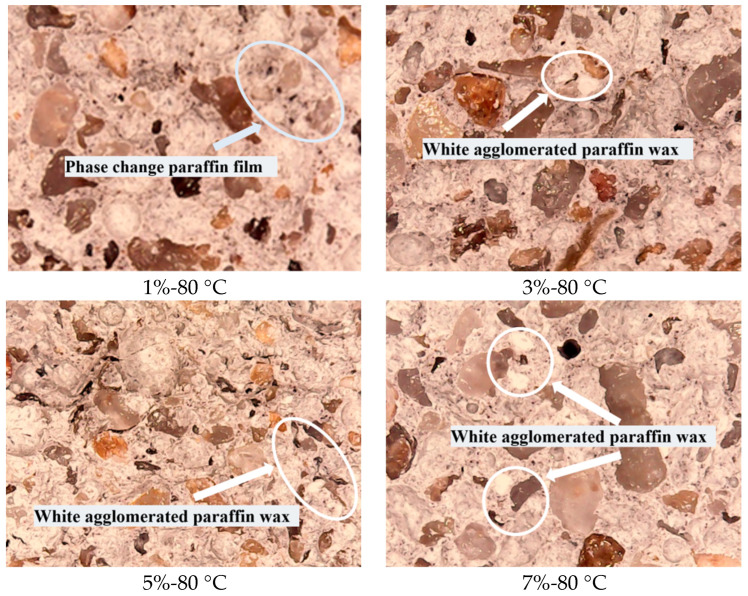
Phase-change melting state of paraffin on the surface of cement mortar under 80 °C.

Figure 7 illustrates the phase-change molten state (50×) of paraffin within specimens cured at 80 °C. The micrograph demonstrates significant reduction in whitish paraffin agglomerates across all dosage levels compared to 60 °C curing. This confirms enhanced phase-change completion in PPFCM with elevated temperatures. Crucially, at 1% paraffin dosage, agglomerates are absent, indicating full phase-change melting. The thoroughly molten paraffin exhibits optimized flowability, diffusing into cementitious pores to form colorless transparent protective films that achieve robust integration with the substrate.

③ Baking temperature 100 °C

**Figure 8:**
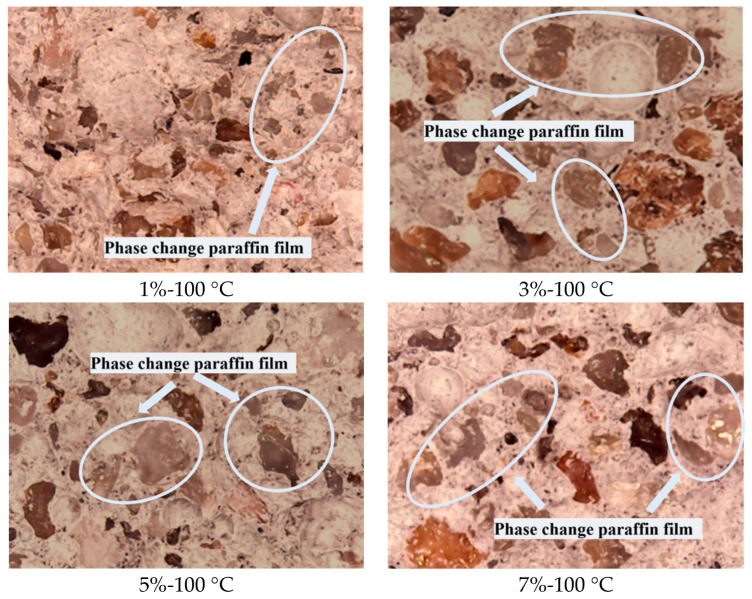
Phase-change melting state of paraffin on the surface of cement mortar under 100 °C.

Figure 8 displays the phase-change molten state (50×) of paraffin in specimens cured at 100 °C. The micrographs show complete elimination of whitish agglomerates across all paraffin dosages, with concurrent emergence of colorless transparent film-structured paraffin. This confirms full phase-change melting throughout the matrix at 100 °C curing temperature. Due to the optical transparency of completely molten paraffin, low-magnification video microscopy fails to resolve morphological details, necessitating SEM for microstructural characterization. Therefore, the phase-change degree of paraffin in PPFCM specimens is positively correlated with baking temperature and negatively correlated with paraffin dosage. However, when the baking temperature reaches high enough (100 °C), the paraffin inside the PPFCM specimens with various paraffin dosage can fully phase into the best film-forming state.

### 3.4. SEM Analysis of Paraffin Phase into Membrane Cement Matrix

**Figure 9:**
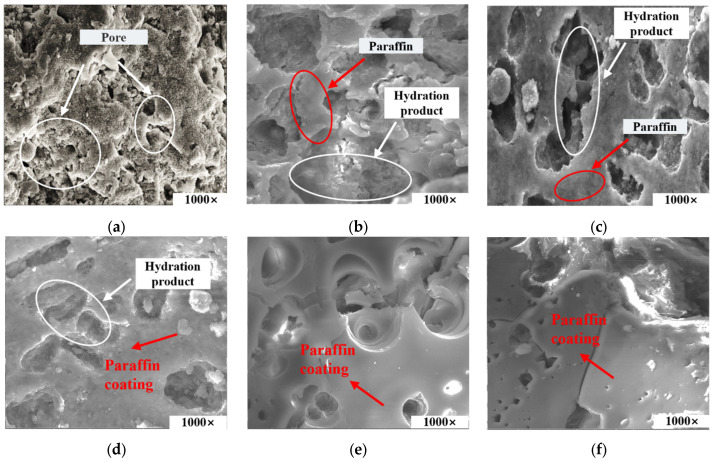
Microscopic morphology of in situ phase-change paraffin in cementitious matrix. (**a**) 0%-Standard curing; (**b**) 1%-60 °C; (**c**) 3%-60 °C; (**d**) 3%-80 °C; (**e**) 3%-100 °C; (**f**) 5%-100 °C.

Figure 9a presents the microstructure (1000×) of plain cement matrix under standard curing. The micrograph reveals abundant hydration products exhibiting a loosely structured, highly porous morphology. This matrix features polydisperse capillary pores with significant depth variations, forming interconnected hydraulic pathways that facilitate water ingress. These intrinsic pore networks constitute the fundamental mechanism underlying hydrophilicity-driven water penetration in conventional cementitious materials.

Figure 9b (1% paraffin, 60 °C curing): The micrograph reveals sparse discrete paraffin domains with limited diffusion radii, indicating incomplete phase-change due to subcritical temperature and low dosage. This results in predominant exposure of hydration products, confirming ineffective film formation. Figure 9c (3% paraffin, 60 °C curing): Increased paraffin accumulation forms localized thickened layers. However, restricted phase-change kinetics prevent continuous film development, maintaining significant hydration product exposure despite higher dosage.

Figure 9d (3% paraffin, 80 °C curing): The micrograph demonstrates enhanced film continuity compared to its 60 °C counterpart at an identical dosage. Elevated temperature significantly improves paraffin diffusion and coalescence, forming near-continuous films. However, suboptimal dosage manifests as cellular morphology with localized depressions where hydration products remain exposed.

Figure 9e (3% paraffin, 100 °C curing): Full thermal activation enables complete paraffin diffusion, forming a consolidated film with significantly reduced defect density. The microstructure exhibits enhanced continuity with only sporadic hydration product exposure at isolated micro-depressions.

Figure 9f shows the micro-morphology of the cement matrix with 5% paraffin dosage at a baking temperature of 100 °C. It can be seen from the figure that when the baking temperature and paraffin dosage are high enough, the paraffin in the cement matrix presents a smooth and dense film structure at the micro-scale, and the cement hydration products are tightly wrapped by these paraffin films, so that no obvious hydration products are exposed. This further indicates that many paraffin particles have fully transformed and diffused to each surface defect of the cement stone to form a film in an under 100 °C baking treatment. Therefore, the phase-change paraffin in the cement matrix presents a dense smooth film-like structure, which firmly adheres to the surface of the pores of the cement matrix, giving PPFCM a strong hydrophobic property.

## 4. Mechanism Analysis

### 4.1. Formation of Phase-Change Film of Paraffin Particles in PPF Cement Matrix

**Figure 10:**
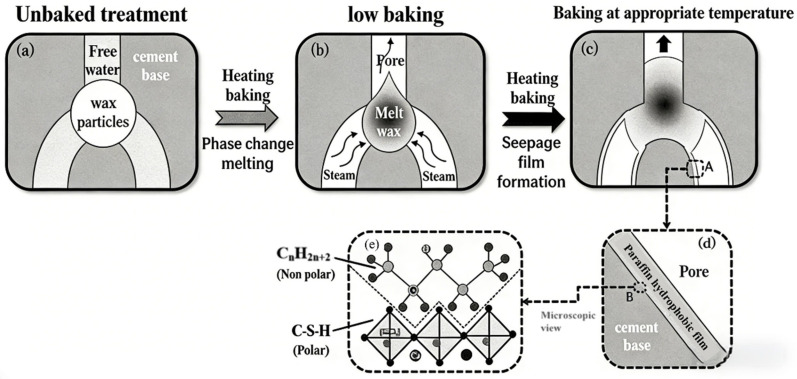
Formation of phase-change film of paraffin particles in PPF cement matrix. (**a**) Unbaked treatment; (**b**) Low baking; (**c**) Baking at appropriate temperature; (**d**) Enlarged view of area A; (**e**) Enlarged view of area B.

The mechanism of phase-change, diffusion, and film formation of paraffin particles within the cementitious matrix is illustrated in Figure 10. As shown in Figure 10a, prior to the curing treatment, each paraffin particle was passively embedded within the cement matrix. No strong integration existed between the paraffin and the surrounding cement paste.

Figure 10b demonstrates that as the curing temperature increased, the paraffin particles began to undergo phase-change under the thermal influence. At lower curing temperatures, the phase-changed paraffin did not fully melt, existing as high-viscosity droplets localized at the original positions of the paraffin particles.

With further elevation of the curing temperature, the degree of melting of the phase-changed paraffin progressively increased, leading to a corresponding enhancement in its fluidity. Simultaneously, the free water within the cement matrix rapidly evaporated into water vapor due to the heat. The accumulation and expansion of this water vapor within the matrix generated an internal vapor pressure directed outward, causing the vapor to exhibit a tendency to diffuse radially. However, the phase-changed paraffin obstructed the pathways for vapor diffusion. Consequently, the pressurized water vapor propelled the molten paraffin to flow outward.

Figure 10c reveals that after sufficient curing at an appropriate temperature, the paraffin underwent complete phase-change, resulting in a fully molten state characterized by low viscosity and high fluidity. Driven physically by the force of water vapor evaporation, this fully molten paraffin gradually permeated into the surrounding capillary pores of the cement matrix. Due to its inherent viscosity, the paraffin continuously adhered to the pore walls during its flow, ultimately forming a robust paraffin film coating.

As depicted in Figure 10d,e, the primary chemical constituents of paraffin are saturated normal alkanes, composed of non-polar long-chain alkane molecules (C_n_H_2n+2_). Consequently, the paraffin film adhering to the pore walls transformed the polarity of the underlying hydration product, C-S-H gel, from its inherent polar state to a non-polar state. This transformation imparted a strong repulsive effect (hydrophobicity) against polar water media. As the molten paraffin fully permeated the surrounding capillary pore network, localized Paraffin Permeation Film (PPF) diffusion zones were ultimately established. The paraffin film within these zones conferred exceptional hydrophobicity and enhanced resistance to aggressive media.

### 4.2. Film-Forming Effectiveness of Paraffin in Cement Matrix

Cement matrices incorporating powdered paraffin were subjected to a baking treatment. During this process, each paraffin particle within the cement matrix began to undergo phase-change under the thermal effect. However, due to the inherently poor thermal conductivity of hardened cement paste, the heat distribution within the cement matrix was non-uniform from the surface to the core. Consequently, achieving the target temperature, particularly in the internal regions of the cement matrix, was challenging.

(1) Curing at 60 °C

The mechanism by which curing temperature and paraffin dosage affect the phase-change and film formation of paraffin within the cement matrix is illustrated in Figure 9. As shown in the figure, at a lower curing temperature (60 °C), the lower internal temperature of the cement matrix resulted in a limited degree of phase-change in the paraffin particles. Specifically, only a minimal surface layer of the particles achieved complete melting and phase-change, while the remaining, insufficiently phase-changed portion persisted as high-viscosity droplets at the original locations of the paraffin particles. Furthermore, at this curing temperature, a higher paraffin dosage (>3%) led to a reduced spacing between adjacent paraffin particles. This caused some insufficiently phase-changed paraffin droplets in close proximity to adhere together, manifesting macroscopically as distinct white agglomerates.

(2) Curing at 80 °C

As the curing temperature increased, the degree of phase-change in the paraffin particles within the cement matrix also increased. Fully phase-changed and molten paraffin exhibited higher fluidity, enabling it to permeate into the surrounding regions under the driving force of water vapor evaporation. This resulted in the formation of broader paraffin diffusion zones around the particles. At a curing temperature of 80 °C, the extent of phase-change in the paraffin particles increased significantly. Only a small core portion of the particles remained insufficiently melted and phase-changed, while the majority of the fully phase-changed paraffin permeated outward, forming larger diffusion zones around the particles. However, if the paraffin dosage was too low (≤3%), the spacing between adjacent particles became excessively large. This prevented the diffusion zones from overlapping, leaving areas of cement hydration products exposed. Consequently, effective hydrophobic modification of the overall cement matrix could not be achieved.

(3) Curing at 100 °C

**Figure 11:**
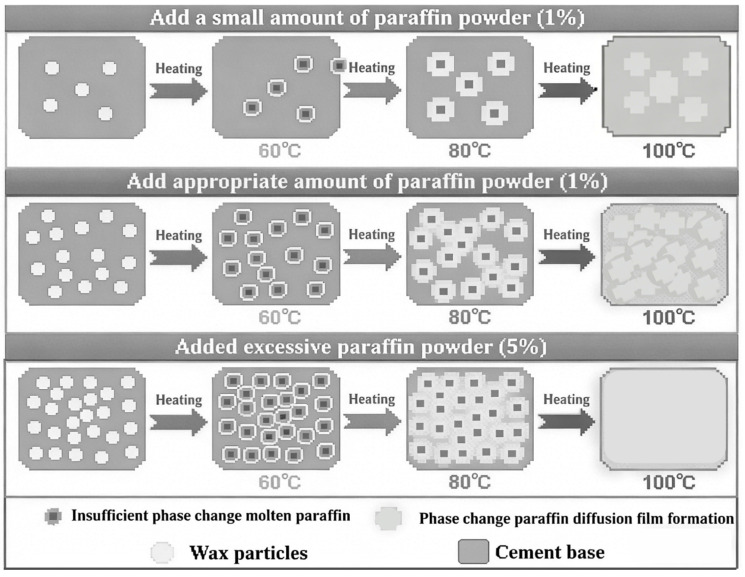
Film-forming effectiveness of paraffin in cement matrix.

At a higher curing temperature (100 °C), the paraffin particles within the cement matrix achieved complete melting and phase-change. As a result, each paraffin particle generated an extensively broad diffusion zone conducive to film formation. Nevertheless, if the paraffin dosage was excessively low (1%), significant gaps remained between the diffusion zones formed around individual particles, making interconnection difficult. As the paraffin dosage increased, the distance between the paraffin diffusion zones progressively decreased. At a higher paraffin dosage (5%), the film-forming diffusion zones around each particle achieved full interconnection, forming an integrated network throughout the cement matrix. This enabled the phase-changed paraffin to form a hydrophobic film at virtually every pore site within the cement matrix, ultimately resulting in comprehensive hydrophobic modification of the cement-based material, as shown in Figure 11.

### 4.3. Enhancement Mechanism of Phase Change Paraffin Film on Carbonation Resistance of Cement-Based Materials

(1) Anti-carbonation mechanism of cement mortar with phase-change-induced film

**Figure 12:**
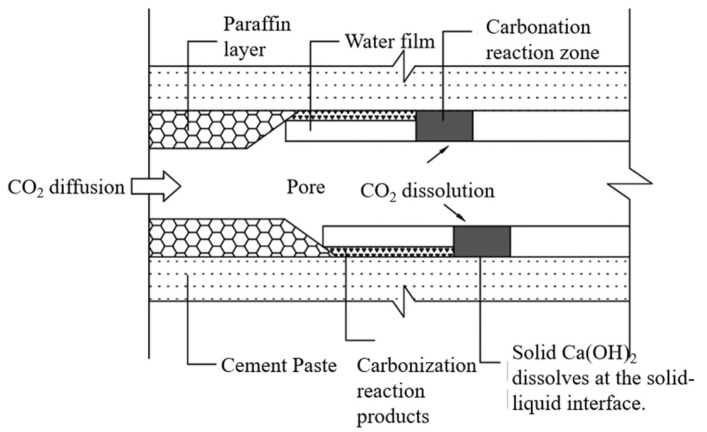
Diagram of carbonization process of mortar with paraffin protective layer.

Even in the area where the paraffin protective layer is most densely distributed, the protective layer cannot completely wrap the surface of the cement stone structure. This not only leaves a diffusion channel for the gas, but also leaves a part of the exposed surface of the cement stone, which is also the reason for the coloration of the phenolphthalein. When the relative humidity and temperature of the environment are constant, the Kelvin diameter dk is constant [1], and the pores smaller than the Kelvin diameter dk and not wrapped in paraffin are gradually filled with water; a layer of water film will be formed on the surface of the pores which are larger than the Kelvin diameter and not wrapped by paraffin (as shown in Figure 12).

On the surfaces of uncoated pores, a thin water film exists. During CO_2_ diffusion, a portion of the gas dissolves into this adsorbed water film. Simultaneously, soluble alkaline components within the hardened cement paste, such as Ca(OH)_2_, gradually dissolve at the solid–liquid interface. Within the liquid phase on the pore surfaces, dissolved Ca(OH)_2_ and other species react with H_2_CO_3_ to form CaCO_3_ and other products. As CO_2_ continuously diffuses and dissolves, the carbonation reaction front progresses inwards.

(2) Influence of paraffin dosage on carbonation performance of cement-based materials

At a paraffin dosage of only 1%, a significant proportion of pores near the surface region of the cement-based material remain uncoated by the paraffin layer. CO_2_ dissolves at the exposed surfaces within this surface region, leading to a high degree of carbonation reaction. Only a small number of pore surfaces enveloped by the paraffin layer remain uncarbonated.

When the paraffin dosage is ≥3%, the hardened cement paste structure within the first 5 mm below the surface is enveloped relatively tightly by the paraffin protective layer. Water vapor can only condense into liquid water within a limited number of capillaries and pores in this zone. Consequently, the reaction in this region is extremely weak during the initial stages of carbonation. Due to the consistently high CO_2_ concentration (20%) maintained in the test environment, CO_2_ that cannot be fully dissolved and reacted in the near-surface region diffuses continuously inwards. The density of the paraffin protective layer gradually decreases with depth. A larger number of pores in the internal regions lack paraffin coating, resulting in the presence of a liquid phase on most pore surfaces. CO_2_ diffusing into these internal regions dissolves and reacts comparatively rapidly. As CO_2_ continuously dissolves and reacts, the alkaline substances within uncoated pores in the zone 0–5 mm below the surface undergo gradual and sustained depletion. Multiple carbonation fronts may potentially coexist simultaneously during the carbonation process of cement-based materials protected by phase-change paraffin.

## 5. Conclusions

(1) With the increase in paraffin dosage, the compressive strength of the modified cement-based materials initially decreased slightly and then declined substantially, with a notable strength reduction detected at a paraffin dosage of 5%. For specimens with the same paraffin content, the compressive strength of cement-based materials with phase-change-induced paraffin films presented a mild downward trend as the curing temperature increased.
(2) The phase-change degree of paraffin embedded in the cement matrix was positively correlated with curing temperature and negatively correlated with paraffin dosage. At a curing temperature of 100 °C, paraffin in the cement matrix could fully melt and complete phase transformation independent of its dosage, forming a colorless, transparent, and well-adhered protective film on the internal pore surface.
(3) The curing-induced phase-change in paraffin enables the formation of a continuous protective film, which effectively encapsulates and blocks the interconnected micro-pore network of cement-based materials. This barrier effect inhibits the ingress of CO_2_ via capillary pores of hardened cement paste, which delivers prominent carbonation resistance under accelerated carbonation conditions. Notably, these findings are derived solely from accelerated carbonation tests; the long-term carbonation resistance and durability of the modified materials require further systematic long-term field and laboratory investigations for comprehensive validation.

## Figures and Tables

**Table 1:** Chemical composition of cement.

SiO_2_	Al_2_O_3_	Fe_2_O_3_	CaO	MgO	f-CaO	Loss
22.1	5.34	3.44	65.3	2.11	0.39	0.13

**Table 2:** Carbonization depth of specimens at different ages (unit: mm).

No.	L0′	L0	L1%	L3%	L5%	L7%	L10%
3d	3.23	3.99	3.43	0	0	0	1.79
7d	6.40	8.59	6.82	4.01	0	0	2.94
28d	8.32	12.25	9.70	5.16	0	2.23	4.83

## Data Availability

The original contributions presented in this study are included in the article. Further inquiries can be directed to the corresponding author.
